# Prediction of clinical outcome and survival in soft-tissue sarcoma using a ten-lncRNA signature

**DOI:** 10.18632/oncotarget.18165

**Published:** 2017-05-22

**Authors:** Rong-quan He, Qing-jun Wei, Rui-xue Tang, Wen-jie Chen, Xia Yang, Zhi-gang Peng, Xiao-hua Hu, Jie Ma, Gang Chen

**Affiliations:** ^1^ Department of Medical Oncology, First Affiliated Hospital of Guangxi Medical University, Nanning, Guangxi Zhuang Autonomous Region, P. R. China; ^2^ Department of Orthopaedics Trauma and Hand Surgery, First Affiliated Hospital of Guangxi Medical University, Nanning, Guangxi Zhuang Autonomous Region, P. R. China; ^3^ Department of Pathology, First Affiliated Hospital of Guangxi Medical University, Nanning, Guangxi Zhuang Autonomous Region, P. R. China; ^4^ Department of Thoracic and Cardiovascular Diseases, First Affiliated Hospital of Guangxi Medical University, Nanning, Guangxi Zhuang Autonomous Region, P.R. China

**Keywords:** soft-tissue sarcoma, lncRNA, prognosis, RNA-seq, TCGA

## Abstract

The prognostic value of long non-coding RNAs (lncRNAs) in patients with soft-tissue sarcoma has rarely been unraveled. The aim of the study was to find a lncRNA signature to predict the clinical outcome and survival in soft-tissue sarcoma based on the high-throughput RNA-seq data from The Cancer Genome Atlas (TCGA) database. The lncRNAs which closely correlated with overall survival in 258 soft-tissue sarcoma patients were identified with Cox proportional regression model. Ten lncRNAs, including RP11-560J1.2, AP001432.14, RP4-665J23.1, LINC00680, AC006129.2, RP11-230G5.2, BACH1-IT2, RP11-274B21.9, RP11-504A18.1 and RP11-713P17.3, were selected to calculate a risk score. The risk score could effectively predict patients’ outcome, such as the status of mitotic count of tumor cells, person neoplasm cancer and residual tumor. More inspiringly, the risk score generated from the 10-lncRNA signature was an independent prognostic indicator for soft-tissue sarcoma patients. Overall, this 10-lncRNA signature gains the potential as an effective prognostic tool for soft-tissue sarcoma as part of the integrated clinical RNA-seq program.

## INTRODUCTION

A carcinoma is a type of cancer arising from epithelial cells. On the contrary, a sarcoma is a malignant tumor originating from all classes of mesenchymal tissues, including cancellous bony, cartilaginous, adipose, muscular, vascular, fibrous or synovial tissues. Hence, the pathology of these sarcomas is particularly varied, which has more than seventy identified subtypes [1–4], Sarcomas are generally divided into two broad categories: osteosarcoma and soft-tissue sarcoma [5, 6].

Unlike carcinoma, sarcomas are rather rare. Basically, sarcomas only account for around 1% of all malignancies in adults and 15% in children. In 2016, a total of 12, 310 cases are estimated to be diagnosed with soft-tissue sarcoma in the United States, with nearly 4, 990 deaths. The lungs are the most common organ for the metastasis of soft-tissue sarcomas, which progresses extremely fast. The tremendous aggressiveness of the soft-tissue sarcomas could partially explain the high mortality rates [3, 7–12].

Though sarcomas are now histopathologically categorized, they have been also molecularly classified into different groups. The molecular advancement has improved the understanding of pathogenesis of soft-tissue sarcomas, as well as provided beneficial targets for clinical settings including diagnosis and treatments [13–19]. However, the pathogenesis of soft-tissue sarcomas is complex due to multiple molecular events being involved. Nowadays, growing evidence has shown that long non-coding RNAs (lncRNAs) play essential roles in the regulation of multiple cellular processes, including tumorigenesis and metastasis of sarcomas; however, the studies available mostly focused on a single lncRNA in osteosarcoma [20–28]. Furthermore, the clinical role of lncRNAs based on high-throughput data has scarcely been investigated. To the best of our knowledge, only Li et al. [29] examined the contributions of lncRNAs to osteosarcoma with microarray analysis. However, no lncRNAs have been studied in soft-tissue sarcomas. Additionally, no investigation on lncRNAs is based on high-throughput sequencing data of soft-tissue sarcomas. To this end, we, for the first time, investigated the clinical value, especially the prognostic role of a series of lncRNAs based on the high-throughput sequencing data from the cancer genomic atlas (TCGA) with 258 cases of soft-tissue sarcomas.

## RESULTS

### Identification of lncRNAs related to overall survival (OS)

According to the exclusion criterion, 258 cases of soft-tissue sarcomas were included in the prognosis analysis. The univariate Cox proportional hazards regression method revealed that a total of 50 lncRNAs gained significant prognostic value for soft-tissue sarcomas with *P* value less than 0.01. Subsequently, multivariate Cox proportional hazards regression analysis was performed to further verify the prognostic roles of these 50 lncRNAs and eventually, a total of 10 lncRNAs (RP11-560J1.2, AP001432.14, RP4-665J23.1, LINC00680, AC006129.2, RP11-230G5.2, BACH1-IT2, RP11-274B21.9, RP11-504A18.1 and RP11-713P17.3) were identified to be independent prognostic indicators of soft-tissue sarcoma. Among these 10 lncRNAs, LINC00680, AC006129.2, RP11-274B21.9 and RP11-713P17.3 were protective biomarkers due to HR being less than one. On the contrary, the other six were risky lncRNAs, since their HRs were all more than one (Table 1).

**Table 1:** The detailed information of ten prognostic lncRNAs significantly associated with overall survival in 258 sarcoma patients

lncRNA	Ensemble ID	Location	β (Cox)	HR (95%CIs)	*P*
RP11-560J1.2	ENSG00000271888	Chromosome 6: 15,243,923-15,245,000	0.582	1.789 (1.269,2.522)	0.001
AP001432.14	ENSG00000242553	Chromosome 21: 37,221,419-37,237,744	0.828	2.288 (1.502,3.480)	0.000
RP4-665J23.1	ENSG00000233593	Chromosome 1: 90,782,983-90,851,657	0.348	1.417 (1.019,1.970)	0.038
LINC00680	ENSG00000215190	Chromosome 6: 57,946,074-57,961,501	−0.681	0.506 (0.310,0.827)	0.007
AC006129.2	ENSG00000268027	Chromosome 19: 41,545,192-41,555,462	−0.236	0.790 (0.657,0.949)	0.012
RP11-230G5.2	ENSG00000250748	Chromosome 12: 65,466,820-65,642,372	0.495	1.641 (1.193,2.256)	0.002
BACH1-IT2	ENSG00000228817	Chromosome 21: 29,370,497-29,373,709	0.423	1.527 (1.046,2.228)	0.028
RP11-274B21.9	ENSG00000271344	Chromosome 7: 128,690,451-128,691,717	−0.557	0.573 (0.420,0.781)	< 0.001
RP11-504A18.1	ENSG00000260971	Chromosome 1: 56,248,294-56,258,571	0.466	1.594 (1.126,2.257)	0.009
RP11-713P17.3	ENSG00000204241	Chromosome 11: 134,032,272-134,046,849	−0.249	0.780 (0.621,0.979)	0.032

### The risk score generated from the 10-lncRNA signature as an independent indicator to predict soft-tissue sarcoma prognosis

**Figure 1:**
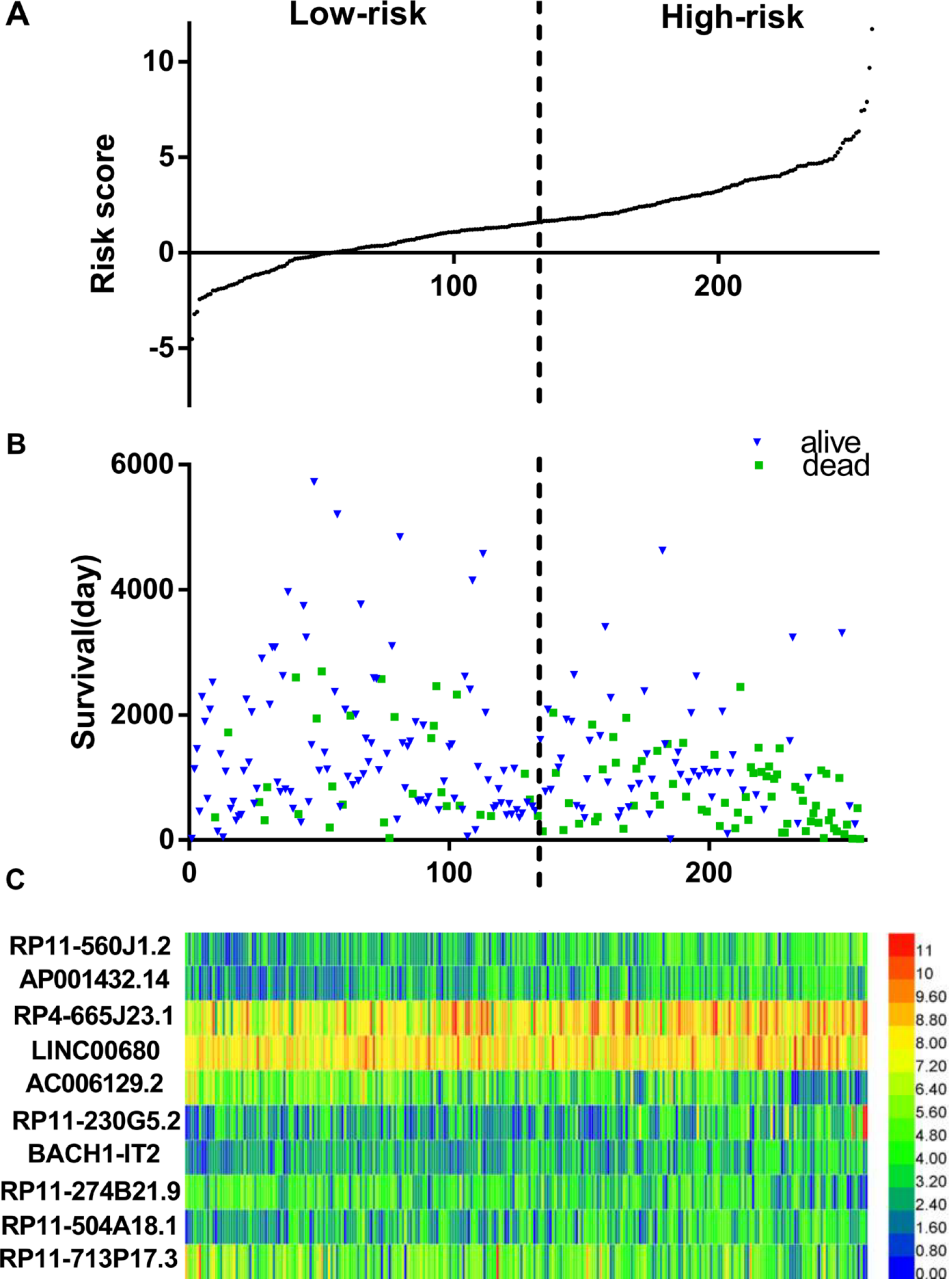
LncRNA predictive risk-score analysis of 258 soft-tissue sarcoma patients in TCGA cohort (**A**) LncRNA risk-score distribution; (**B**) Patients survival; (**C**) Heatmap of lncRNA expression profiles of sarcoma patients. The black dotted line represents the median signature cutoff dividing patients into low-risk and high-risk groups.

**Figure 2:**
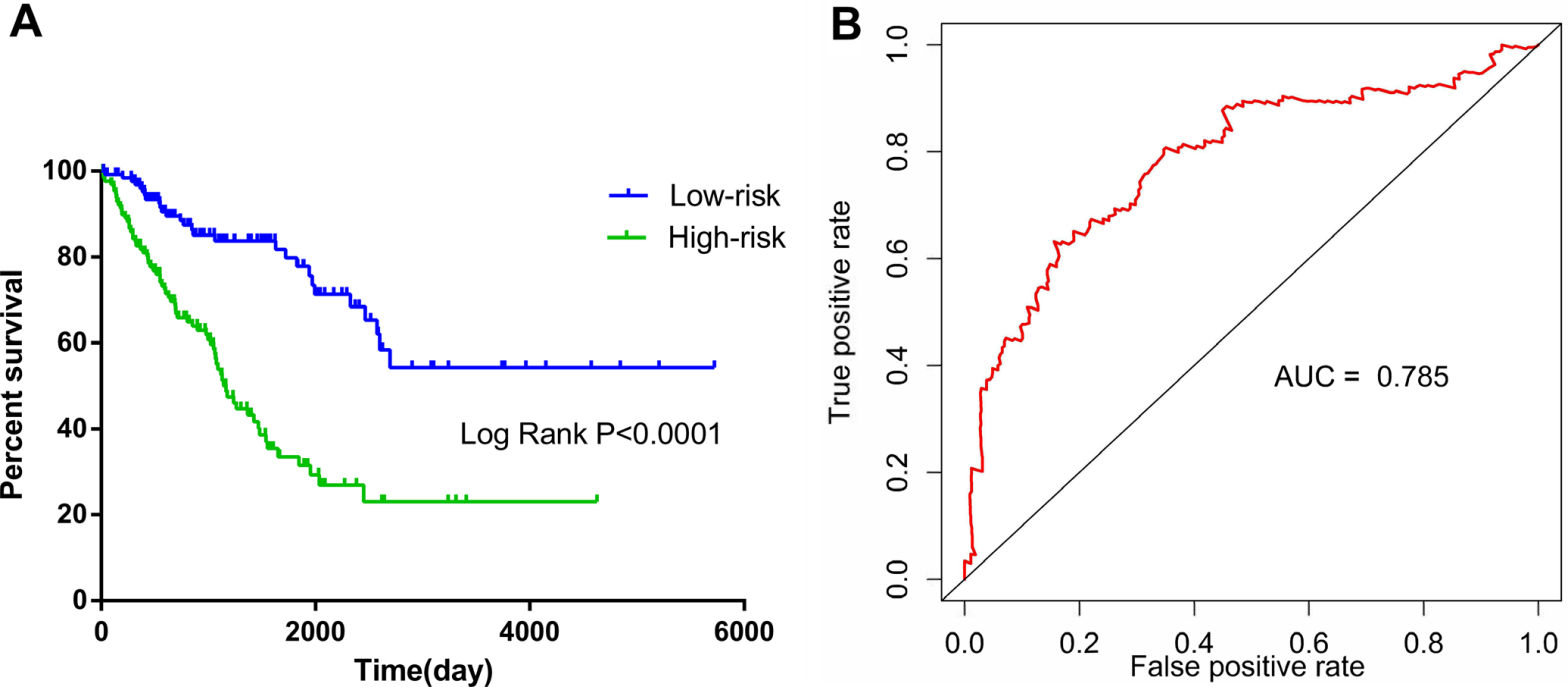
Kaplan–Meier and survival ROC curves for the ten-lncRNA signature in TCGA soft-tissue sarcoma cohort (**A**) The Kaplan – Meier curves for high-risk and low-risk group sarcoma patients from the TCGA cohort divided by the median cutoff point. (**B**) The ROC curve had an AUC of 0.785.

The risk score for predicting the OS of soft-tissue sarcoma prognosis was generated with the formula based on the 10 lncRNAs above according to previous reports [30–32]. The risk score generated from the 10-lncRNA signature = expression of RP11-560J1.2 * 0.582+ expression of AP001432.14 * 0.828 + expression of RP4-665J23.1 * 0.348 + expression of LINC00680 * −0.681 + expression of AC006129.2 * −0.236 + expression of RP11-230G5.2 * 0.495 + expression of BACH1-IT2 * 0.423 + expression of RP11-274B21.9 * −0.557 + expression of RP11-504A18.1 * 0.466 + expression of RP11-713P17.3 * -0.249. Each soft-tissue sarcoma patient had a score based on the formula above and all patients were then divided into two groups: low-risk (*n* = 129) and high-risk (*n* = 129) on the basis of the median point of the prognostic risk score (Figure 1A). The survival status of each patient was shown in Figure 1B and a heatmap was drawn to display the expression level of the top 10 lncRNAs for each patient (Figure 1C). More importantly, the risk score could act as an independent indicator for the OS of soft-tissue sarcomas, as the HR was 1.445 (95% CI: 1.321–1.581, *P* < 0.001) assessed by the univariate Cox regression analysis and K-M method (Table 2, Figure 2).

**Table 2:** Univariate Cox regression analysis of overall survival in each cohort

Clinical features	Number	*P*	HR	95%CIs of HR	
Risk score (High-risk/Low-risk)	129/129	< 0.001	1.445	1.321	1.581
Gender(male/female)	118/140	0.439	1.172	0.784	1.751
Age(< = 60/> 60)	128/130	0.134	1.357	0.91	2.023
Leiomyosarcoma histologic subtype(poorly differentiated or pleomorphic or epithelioid leiomyosarcoma/conventional leiomyosarcoma/well-differentiated leiomyosarcoma (resembling leiomyoma)	34/64/4	0.025	0.592	0.303	0.924
leiomyosarcoma major vessel involvement(yes/no)	12/78	0.483	0.685	0.239	1.968
New neoplasm event type(new primary tumor /distant metastasis/locoregional recurrence)	5/41/29	0.668	0.885	0.506	1.548
Local disease recurrence(yes/no)	29/143	0.002	2.316	1.37	3.916
Metastatic diagnosis(yes/no)	56/119	< 0.001	3.009	1.831	4.946
Tumor depth(deep/superficial)	184/21	0.069	2.924	0.921	9.285
Contiguous organ invaded(yes/no)	14/43	0.012	2.809	1.261	6.261
Margin status(positive/negative)	73/136	0.013	1.84	1.138	2.974
Person neoplasm cancer status(with tumor/tumor free)	124/124	< 0.001	9.532	5.07	17.92
Residual tumor(yes/no)	154/78	< 0.001	2.553	1.668	3.909
Tumor total necrosis percent(0/< 10%/> = 10%,< 50%	12/61/38	0.121	1.207	0.952	1.532
Radiation therapy(yes/no)	73/179	0.508	0.882	0.566	1.376
Treatment completion success outcome(SD/PD/CR/PR)	124/3/8/64	< 0.001	2.386	1.795	3.171

**Figure 3:**
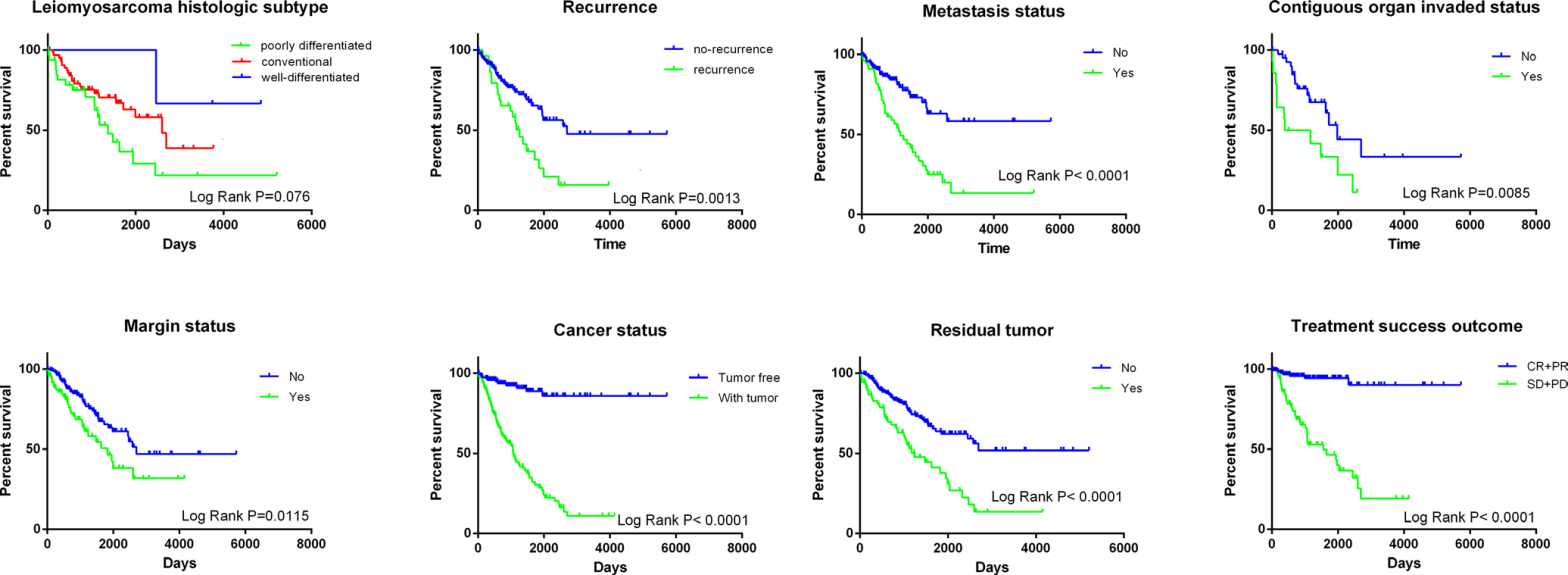
Kaplan–Meier curves of clinical features for the overall survival of soft-tissue sarcoma patients

The prognostic role of the risk score was also compared to the classical clinicopathological parameters of soft-tissue sarcoma (Table 2). Among 199 patients with complete clinical data, eight (4.0%) achieved complete response (CR), 64 (32.2%) partial response (PR), 124 (62.3%) stable disease (SD) and three (1.5%) progressive disease (PD). The median age for all patients was 60 years old. The histopathological subtypes of these 258 soft-tissue sarcomas included 103 leiomyosarcomas (LMS) (39.9%), 58 dedifferentiated liposarcomas (22.5%), 51 undifferentiated pleomorphic sarcomas (19.8%), 25 myxofibrosarcomas (9.7%), 10 synovial sarcomas (3.9%), 9 malignant peripheral nerve sheath tumors (MPNST) (3.5%) and 2 desmoid tumors (0.8%). Univariate cox regression analysis of OS showed that several clinicopathological parameters could predict the survival of soft-tissue sarcomas, including the histological subtype, local disease recurrence, metastasis, invasion of contiguous organ, margin status, person neoplasm cancer status, residual tumor and treatment completion success outcome (Table 2, Figure 3). Not surprisingly, the “person neoplasm cancer status” was the most remarkable parameter to predict survival and patients with tumor were 9.532 times more prone to suffer from death than those who were tumor free.

### The risk score of the 10-lncRNA-signature related to the progression and treatment outcome of soft-tissue sarcomas

**Figure 4:**
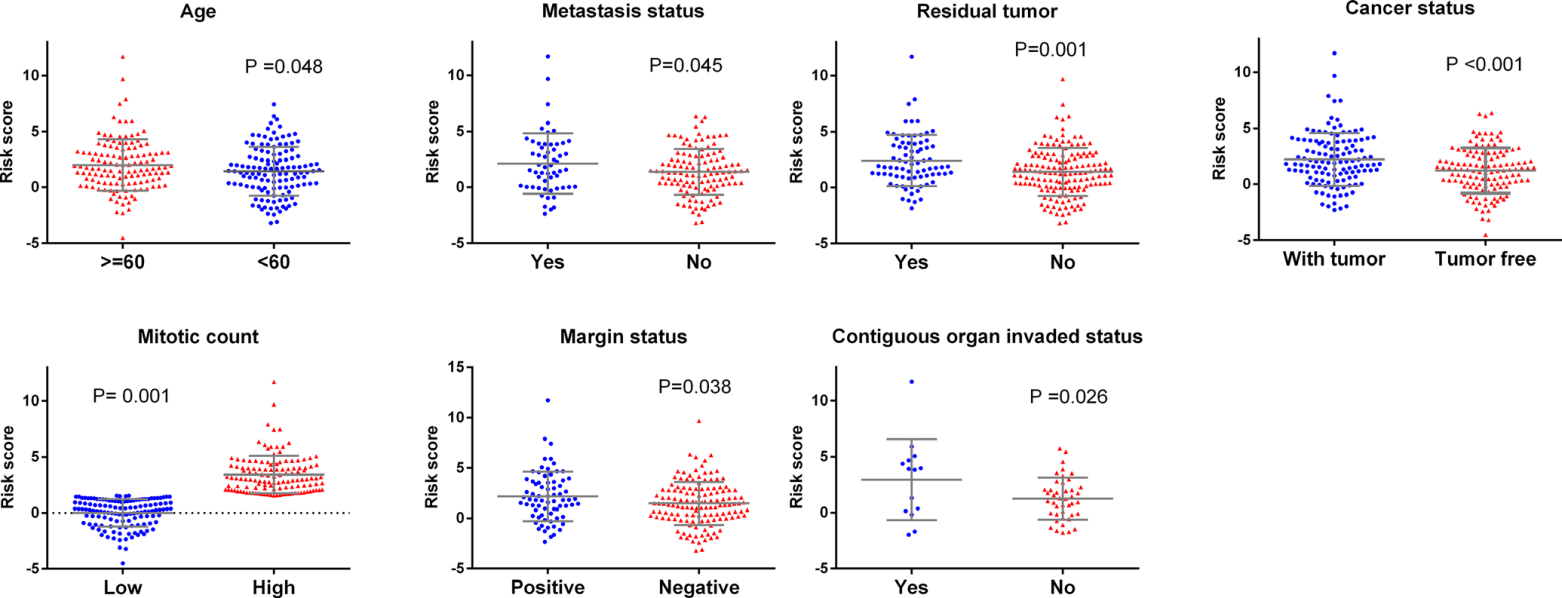
Relationship between clinical features and risk score (**A**) Age; (**B**) Metastasis status (**C**) Residual tumor (**D**) Tumor status (**E**) Mitotic count; (**F**) Margin status; (**G**) Contiguous organ invaded.

**Figure 5:**
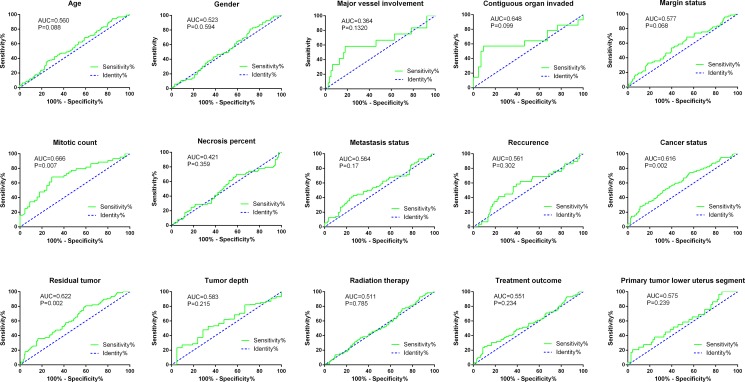
ROC curves of risk score for clinical features in soft-tissue sarcoma patients

The relationship between the risk score of the 10-lncRNA-signature and different clinical features was also analyzed. The student's *t* test disclosed that the risk score clearly varied between two groups of different ages (*P* = 0.048), contiguous organ invaded status (*P* = 0.026), margin status (*P* = 0.038), mitotic count (*P* = 0.001), metastasis status (*P* = 0.045), person neoplasm cancer status (*P* < 0.001) and residual tumor (*P* = 0.001, Supplementary Table 1, Figure 4). Furthermore, the ROC curves indicated that the risk score could largely predict the status of mitotic count, person neoplasm cancer and residual tumor with the AUCs being 0.666, 0.616 and 0.622, respectively (Figure 5). The Spearman Correlation test also confirmed the close relationships between risk score and mitotic count (*r* = 0.297, *P* = 0.005), person neoplasm cancer status (*r* = 0.201, *P* = 0.001) or residual tumor (*r* = 0.2, *P* = 0.002, Supplementary Table 1).

**Figure 6:**
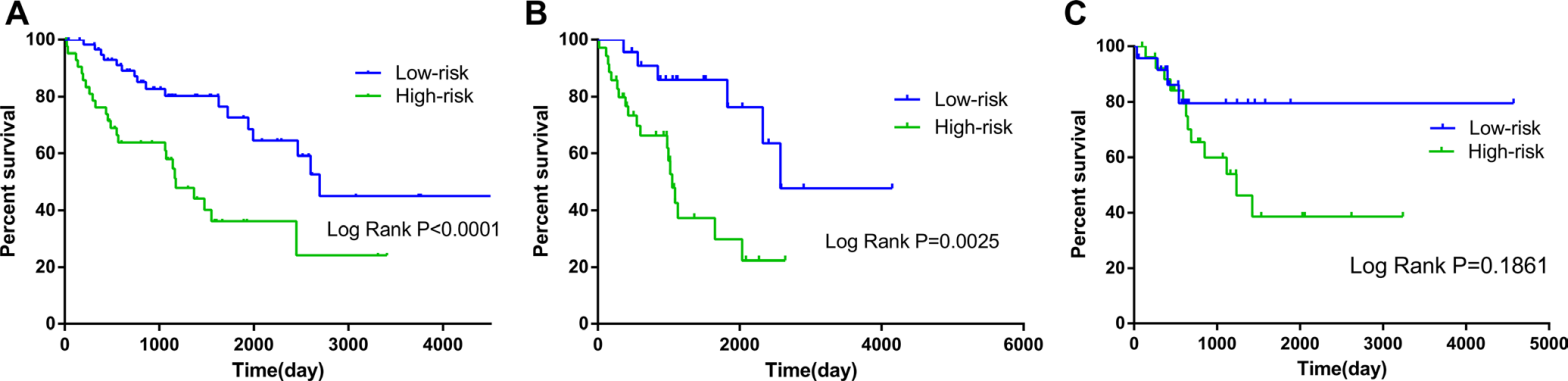
Kaplan–Meier curves for prognostic value of risk-score signature according to histologic subtypes (**A**) Dedifferentiated liposarcoma (**B**) Leiomyosarcoma (LMS); (**C**) Undifferentiated sarcoma.

**Figure 7:**
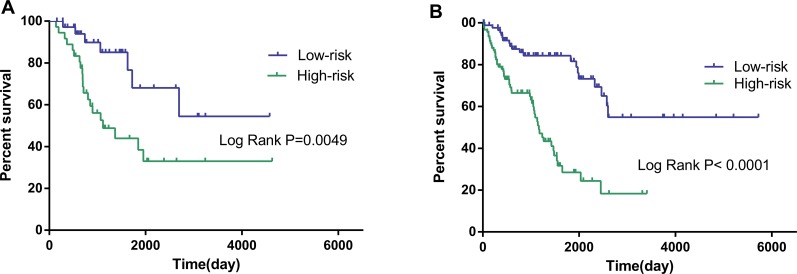
Kaplan–Meier curves for prognostic value of risk-score signature for the patients divided by treatment modalities (**A)** Radiation therapy (**B**) Non-radiation therapy.

The risk score was notably different among all soft-tissue sarcomas of different histology, as well as among distinct differentiation levels in leiomyosarcoma (Supplementary Table 1). When the patients were stratified into different subgroups based on differentiation, the risk score of the 10-lncRNA signature remained to be an independent prognostic indicator for the subgroups of dedifferentiated liposarcoma and leiomyosarcoma (Table 3, Figure 6). According to the National Comprehensive Cancer Network (NCCN) Guidelines (Soft Tissue Sarcoma, https://www.nccn.org), radiation therapy influences the survival of soft-tissue sarcoma. In consideration of this, we also adjusted radiation therapy as a parameter to better reveal the prognostic value of the 10-lncRNA-based risk score for overall survival. No matter radiation therapy was received or not, the 10-lncRNA signature was a stable prognostic marker for soft-tissue sarcoma patients (Table 3, Figure 7).

**Table 3:** Prognostic value of risk score stratified by histological type and treatment modality

Parameters	Number	HR	95% CIs		*P*
**Histological type**					
Leiomyosarcoma (LMS)	103	2.901	1.538	5.473	0.001
Dedifferentiated liposarcoma	58	3.952	1.529	10.211	0.005
Undifferentiated pleomorphic sarcoma	51	2.116	0.679	6.596	0.196
**Treatment modality**					
Radiation therapy	178	3.865	2.267	6.588	< 0.001
Non-radiation therapy	73	3.082	1.298	7.318	0.011

Since the most life-threatening aspect of soft-tissue sarcomas is their capacity to disseminate hematogenously. We were also interested in the effect of each lncRNA on the metastasis. However, there was no significant difference of the level of these lncRNAs between metastatic and non-metastatic sarcomas. Regarding to ROC analysis, only RP4-665J23.1 conferred a mild diagnostic value for metastasis in soft-tissue sarcomas (AUC=0.615, *P* = 0.0146, Supplementary Table 2 in Supplementary Information).

### Potential functional assessment of the lncRNAs by multi experiment matrix

**Figure 8:**
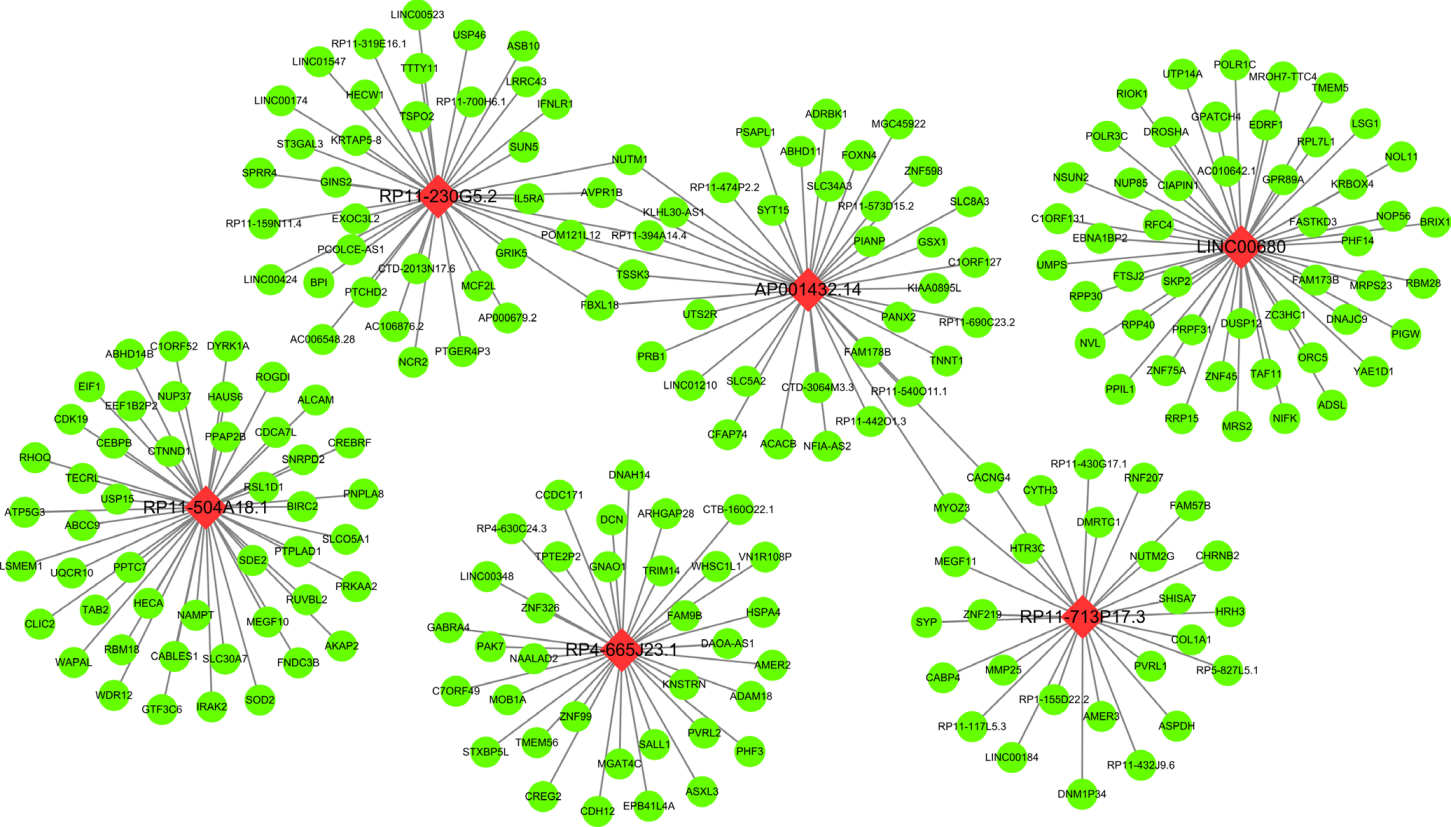
Regulation network of each key lncRNA by multi experiment matrix This network was established based on the top 50 target genes for the lncRNA by utilizing the Multi Experiment Matrix. The green balls present the target genes and the red diamonds show the key lncRNAs.

**Figure 9:**
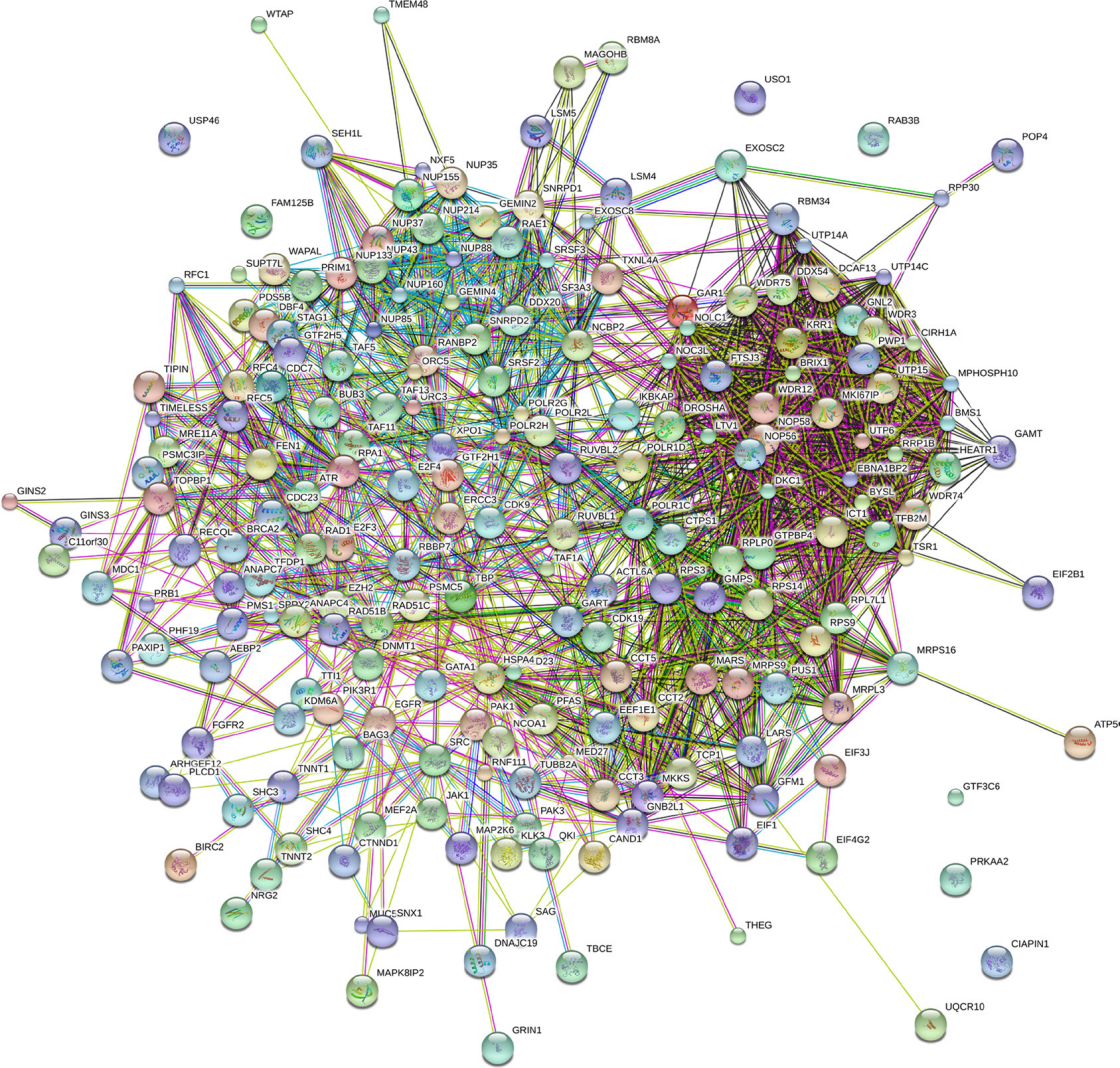
The protein protein interaction of correlative genes for the lncRNAs The protein-to-protein network analysis was performed using STRING (version: STRING 10.0).

Among these ten lncRNAs (four protective lncRNAs: LINC00680, AC006129.2, RP11-274B21.9 and RP11-713P17.3 and six risky lncRNAs: RP11-560J1.2, AP001432.14, RP4-665J23.1, RP11-230G5.2, BACH1-IT2, and RP11-504A18.1), the relevant genes of key lncRNAs were evaluated by MEM, and the co-expression networks of lncRNAs was visualized by MEM (Figure 8, Supplementary Figures 1–6 in Supplementary Information). Due to the absence of valid probe sets for four lncRNAs (RP11-560J1.2, AC006129.2, BACH1-IT2, RP11-274B21.9) on Affymetrix Gene Chip Human Genome U133 Plus 2.0 Array platform, we could obtain only six of ten lncRNAs from this online tool (AP001432.14, RP4-665J23.1, LINC00680, RP11-230G5.2, RP11-504A18.1, RP11-713P17.3), thus only these six lncRNAs were presented. We performed additional protein–protein interaction analysis for all the genes significantly co-expressed with the lncRNAs and found out three hub genes (RANBP2, POLR1B, GMPS) (Figure 9). The correlative genes of each lncRNA were enriched in multiple pathways assessed by GO and KEGG analyses (Supplementary Table 3).

## DISCUSSION

Soft-tissue sarcoma represents a rare cancer entity, accounting for less than 1% of adult malignancies. The keystone of curative intent treatment is surgery with free margins. However, multimodal methodologies which often entail radiation therapy have substituted extensive surgical procedures to preserve functionality while maintaining sufficient local control [33–37]. In terms of the aggressiveness and shortness of specific biomarkers for soft tissue sarcoma patients, there is a crucial requirement for trustworthy prognostic indicators identifying a subclass of patients with poor prognosis, who would hence benefit from extra treatment possibilities [38, 39]. Recently, large-scale genomic analyses have shown a set of molecular events were closely related to the tumorigenesis or progression of soft-tissue sarcomas. Subramanian et al. [40] conducted a comprehensive investigation into microRNA expression signatures of 27 cases of sarcomas, 5 cases of normal smooth muscle and 2 cases of normal skeletal muscle tissues with microarray and small RNA cloning approaches. The identification of exceptional microRNA signatures in every sarcoma type may specify their function in pathogenesis of soft-tissue sarcomas.

However, all existing studies on the relationship between lncRNA and soft-tissue sarcomas focused on single lncRNA and single disease. For example, higher expression of lncRNA HOTAIR was detected in both primary and metastatic sarcoma tissues. High level expression of HOTAIR in the primary sarcoma was also significantly related to metastasis. HOTAIR expression level tend to be correlated with the necrotic status in various sarcoma tissues [41]. But no studies have been performed so far to investigate the clinical role of an lncRNA signature in soft-tissue sarcoma based on high-throughput data. In the current study, a comprehensive investigation of lncRNA expression based on RNA-seq data was conducted with a large cohort of soft-tissue sarcoma patients from the data of TCGA database, including leiomyosarcoma, dedifferentiated liposarcoma, undifferentiated pleomorphic sarcoma, myxofibrosarcoma synovial sarcoma and malignant peripheral nerve sheath tumors. Ten lncRNAs showed the most potent prognostic value, including four protective lncRNAs (LINC00680, AC006129.2, RP11-274B21.9 and RP11-713P17.3) and six risky lncRNA: RP11-560J1.2, AP001432.14, RP4-665J23.1, RP11-230G5.2, BACH1-IT2, and RP11-504A18.1). More importantly, the risk score calculated by the 10-lncRNA signature was capable to predict the outcome and overall survival of soft-tissue sarcoma independent of other clinicopathological features, which was assessed by Cox regression analysis. This novel 10-lncRNA signature could be an independent prognostic indicator for soft-tissue sarcoma and this study discovered the potency of a combined lncRNA signature to properly predict the survival of soft-tissue sarcoma.

We attempted to validate the current findings from Gene Expression Omnibus (GEO, https://www.ncbi.nlm.nih.gov/gds) and ArrayExpress (http://www.ebi.ac.uk/arrayexpress/). “soft-tissue sarcoma” OR “soft tissue sarcoma” OR leiomyosarcoma OR liposarcoma OR “undifferentiated pleomorphic sarcoma” OR myxofibrosarcoma OR “synovial sarcoma” OR “peripheral nerve sheath tumor” were searched and altogether 278 series were achieved (data not shown). Next, we searched our 10 lncRNAs in these series and unfortunately, only GSE21050 could be included with the data of AC006129.2 and RP11-504A18.1. But no survival data was available and we could only assess the relationship between the level of lncRNAs and metastasis. No significant correlations between these 2 lncRNAs and metastasis could be noted (Supplementary Figures 7, 8 in Supplementary Information). So our findings from TCGA need to be verified by other experiments in the future.

Among all the 10 lncRNAs to calculate the risk score, none of researchers has reported in literatures so far. Hence, the clinical role or biological function of any of them remains absolutely unknown. Previous studies have proposed that lncRNAs may take part in different biological processes by interrelating with correlative genes. To this end, MEM was used to gather the correlative genes of each lncRNA and also to inspect the rough molecular mechanism. The results suggested that the six of these 10 prognostic lncRNAs might be related to a number of genes and multiply signaling pathways involved in soft-tissue sarcoma. Even these lncRNAs have consistent prognostic value for soft-tissue sarcoma, they may share absolutely different molecular mechanisms. Interestingly, from KEGG analysis, the Neuroactive ligand-receptor interaction pathway and Calcium signaling pathway are the top 2 pathways for both AP001432.14 and RP11-230G5.2, indicating that these 2 lncRNAs may have similar functions via targeting comparable signaling pathways; however, validation with additional *in vitro* and *in vivo* experiments is requisite to uncover the underlying molecular mechanism of lncRNAs in soft-tissue sarcoma.

By mining the high throughput RNA-seq and clinical data from TCGA database, we identified a specific 10-lncRNA signature closely related to patient survival in soft-tissue sarcoma that can provide a potent prognostic tool for this class of tumors. However, the current finding based on a single cohort of TCGA needs further validation by other detecting methods, for instance, real time RT-qPCR and fluorescence *in situ* hybridization. Furthermore, the biological function and molecular mechanism of these lncRNAs remain unexplored, which need to be explored in-depth in the future.

## MATERIALS AND METHODS

### Patient information and lncRNA expression profiles

The mRNA expression data (Level 3) and clinical data for soft-tissue sarcoma patients (up to January 22, 2017) were achieved from TCGA data portal. The expression of 7549 lncRNAs in soft-tissue sarcoma samples was analyzed on IlluminaHiSeq mRNA Seq platform. Those samples without lncRNA sequence data or clinical data were omitted. Therefore, a sum of 258 soft-tissue sarcoma patients were involved in the current study, with matching clinical data including gender, age, histology, differentiation, leiomyosarcoma major vessel involvement, new neoplasm event type, local disease recurrence, metastatic diagnosis, tumor depth, contiguous organ invaded, margin status, person neoplasm cancer status, residual tumor, tumor total necrosis percent, radiation therapy and treatment completion success outcome (SD/PD/CR/PR) were involved in the current study (Table 2, Supplementary Table 1). The end-point was OS in this study for the soft-tissue sarcoma. As the data were downloaded from TCGA, additional approval by ethics committee was not obligatory. Data was processed according to the TCGA human subject protection and data access policies.

### Statistical analysis

The expression level of 7549 lncRNAs was shown as raw count lncRNA mapped data. The lncRNAs which were less than 1 raw count in exceeded 10% of all subjects were eliminated using R language. The expression level of each lncRNA was log2 transformed for further analysis. The univariate Cox proportional hazards regression with significance level set as 0.01 was performed to find out the lncRNAs evidently associated with OS. The filtered lncRNAs were divided into risky (with a hazard ratio (HR) for death greater than 1) and protective (based on a HR for death less than 1) types. A risk score formula for predicting OS was developed based on a linear combination of the expression level that multiplied regression coefficient derived from the multivariate cox stepwise regression model (β): risk score = expression of gene 1*β gene 1 + expression of gene 2*β gene 2 + …expression of gene n*β gene n. The “β” value is the estimated regression coefficient of lncRNA derived from the multivariate Cox stepwise regression analysis. By utilizing the median risk score as the cutoff point, the soft-tissue sarcoma patients were divided into two subgroups of high score and low score. We also used the R package “survivalROC” to assess the predictive accuracy of prognostic model for time dependent disease outcomes within 5 years as the defining point.

Univariate Cox proportional hazards regression analyses were performed to explore the effects of clinical features and the risk score on OS of soft-tissue sarcoma patients. Each predictor identified via univariate analysis was further assessed by multivariate cox proportional hazards regression analysis. Survival curves were generated by the Kaplan-Meier and log-rank method. The difference of risk score between diverse groups according to various clinical parameters was assessed by student *t* test or ANOVA test. ROC curves were used to evaluate the predictive values of risk score for different parameters representative of patients’ outcome and survival. The Spearman Correlation test was conducted to evaluate the correlation between risk score and the progression of soft-tissue sarcoma. The student *t*-test, ROC analysis as well as Spearman correlation test were also performed to examine the relationship between these novel lncRNAs and metastasis individually, since metastasized soft-tissue sarcomas generally indicate a poorer prognosis. Statistical significance was defined as a two-sided *P* value < 0.05. The statistical analyses were performed with SPSS22.0 software.

### Potential functional assessment of the lncRNAs by multi experiment matrix

Then, we also explored the co-expressed genes for the key lncRNAs by Multi Experiment Matrix (MEM, http://biit.cs.ut.ee/mem/index.cgi) based on Affymetrix Gene Chip Human Genome U133 Plus 2.0 Array platform [42, 43]. After the identification of weighted correlation, Cytoscape 3.4.0 was used to show the network between lncRNAs and their related genes. GO and KEGG analyses were also performed based on the Database for Annotation, Visualization and Integrated Discovery (DAVID, https://david.ncifcrf.gov/). The protein-to-protein network analysis was conducted by STRING (version: STRING 10.0).

## SUPPLEMENTARY TABLES AND FIGURES






