# Supplementary material for: Prediction of clinical outcome and survival in soft-tissue sarcoma using a ten-lncRNA signature
Source: Oncotarget. 2017 May 22;8(46):80336–47. doi: 10.18632/oncotarget.18165 (PMC5655202; doi:10.18632/oncotarget.18165)
Supplement: Supplementary file 1 [file oncotarget-08-80336-s001.pdf]

Prediction of clinical outcome and survival in soft-tissue sarcoma using a ten-lncRNA signature

SUPPLEMENTARY MATERIALS

Supplementary Table 1: The association of the risk score of the ten-lncRNA signature with clinical features in soft-tissue sarcoma patients. See Supplementary_Table_1

Supplementary Table 2: The association between the lncRNAs and metastasis in sarcoma patients

LncRNAs	Metastasis	*t*-test					ROC			Spearman	
		n	**mean**	**SD**	***t***	***P***	**AUC**	**95%CIs**	***P***	***r***	***P***
RP11-560J1.2	no	119	3.39	1.385	−1.186	0.237	0.555	0.478–0.630	0.2242	0.092	0.228
	yes	56	3.66	1.379							
AP001432.14	no	119	3.1	1.362	−0.282	0.778	0.505	0.429–0.582	0.9052	0.009	0.907
	yes	56	3.16	1.187							
RP4-665J23.1	no	119	7.84	1.6	−1.553	0.122	0.615	0.538–0.687	0.0146	0.189	0.012
	yes	56	8.27	1.892							
LINC00680	no	119	8.24	1.006	−0.884	0.378	0.543	0.466–0.619	0.326	0.074	0.333
	yes	56	8.38	0.906							
AC006129.2	no	119	4.58	1.875	1.205	0.23	0.55	0.473–0.625	0.2847	−0.082	0.283
	yes	56	4.21	1.866							
RP11-230G5.2	no	119	2.94	1.633	−1.172	0.245	0.539	0.462–0.614	0.4213	0.064	0.399
	yes	56	3.38	2.534							
BACH1-IT2	no	119	2.99	1.108	0.351	0.726	0.513	0.436–0.589	0.7799	−0.021	0.779
	yes	56	2.93	1.11							
RP11-274B21.9	no	119	3.94	1.304	0.697	0.487	0.529	0.452–0.605	0.5393	−0.048	0.526
	yes	56	3.79	1.522							
RP11-504A18.1	no	119	3.17	1.317	−0.526	0.6	0.552	0.475–0.627	0.2698	0.087	0.254
	yes	56	3.29	1.51							
RP11-713P17.3	no	119	4.84	1.813	0.943	0.347	0.527	0.450–0.603	0.573	−0.044	0.56
	yes	56	4.55	2.008							

Supplementary Table 3: Functional enrichment analyses of the protein-coding genes co-expressed with the six prognostic lncRNAs. See Supplementary_Table_3

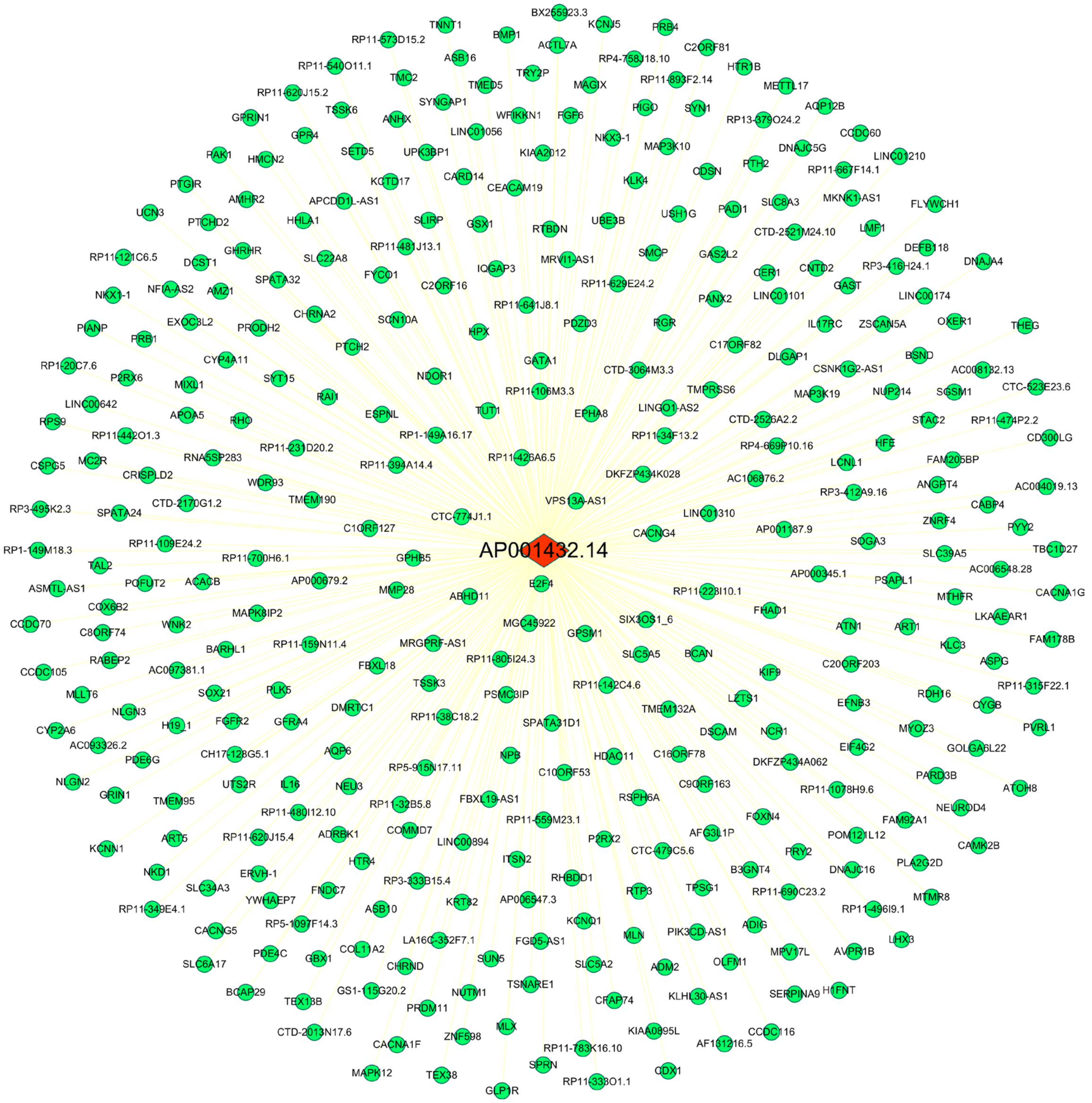

Supplementary Figure 1: Regulation network of lncRNA AP001432.14 by multi experiment matrix.

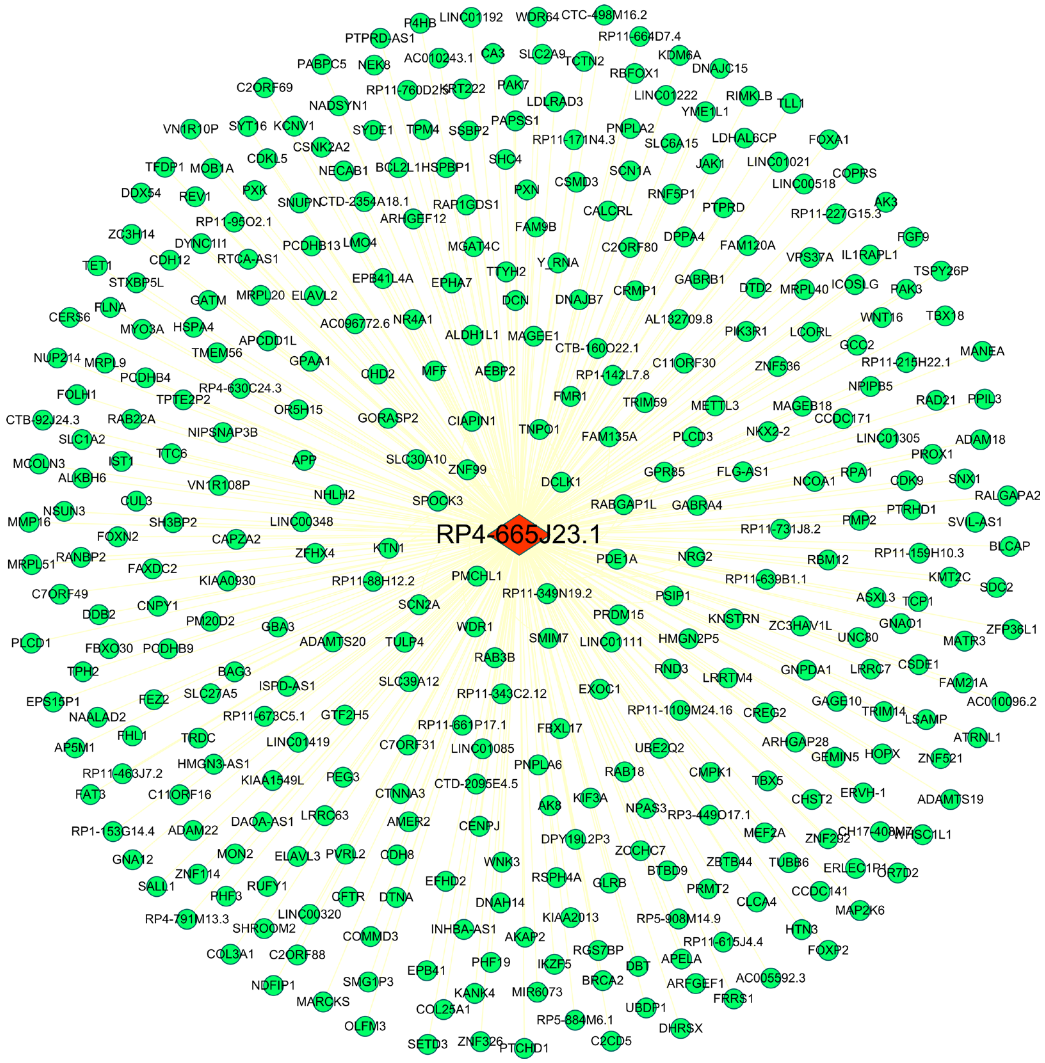

Supplementary Figure 2: Regulation network of lncRNA RP4-665J23.1 by multi experiment matrix.

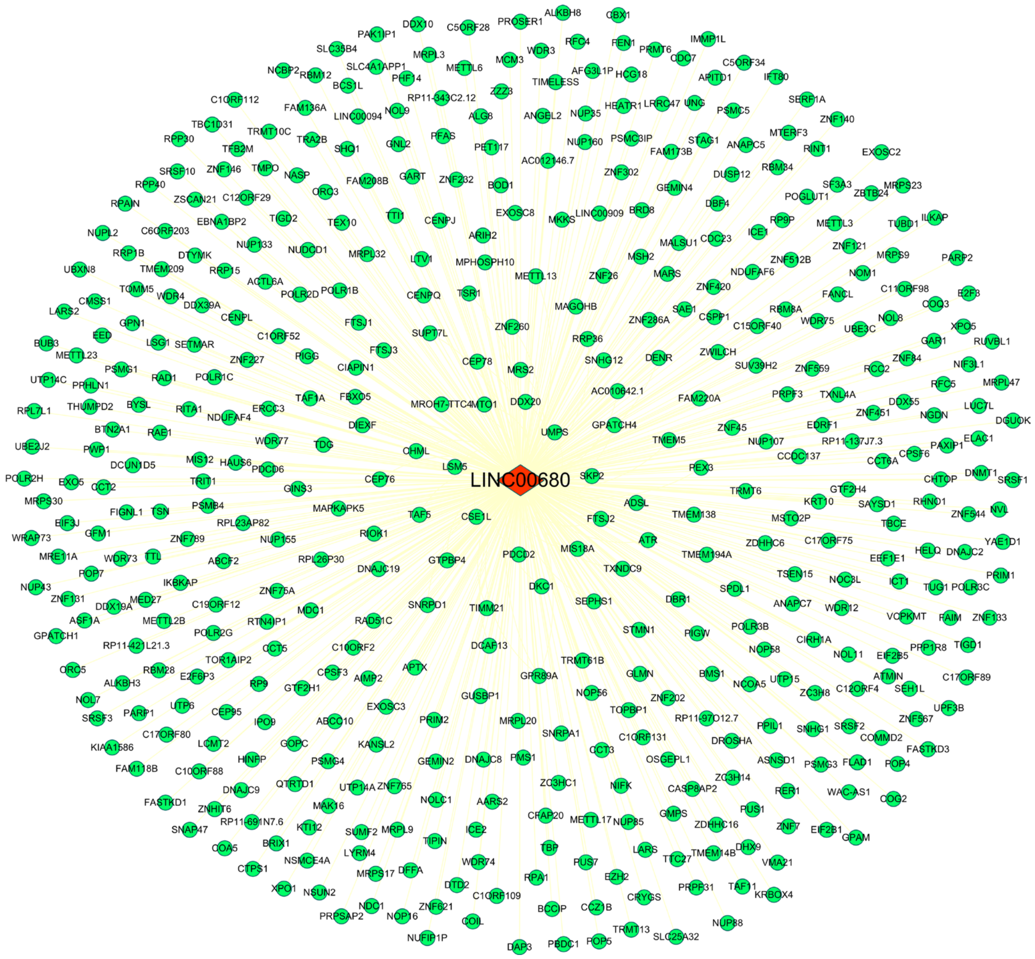

Supplementary Figure 3: Regulation network of lncRNA LINC00680 by multi experiment matrix.

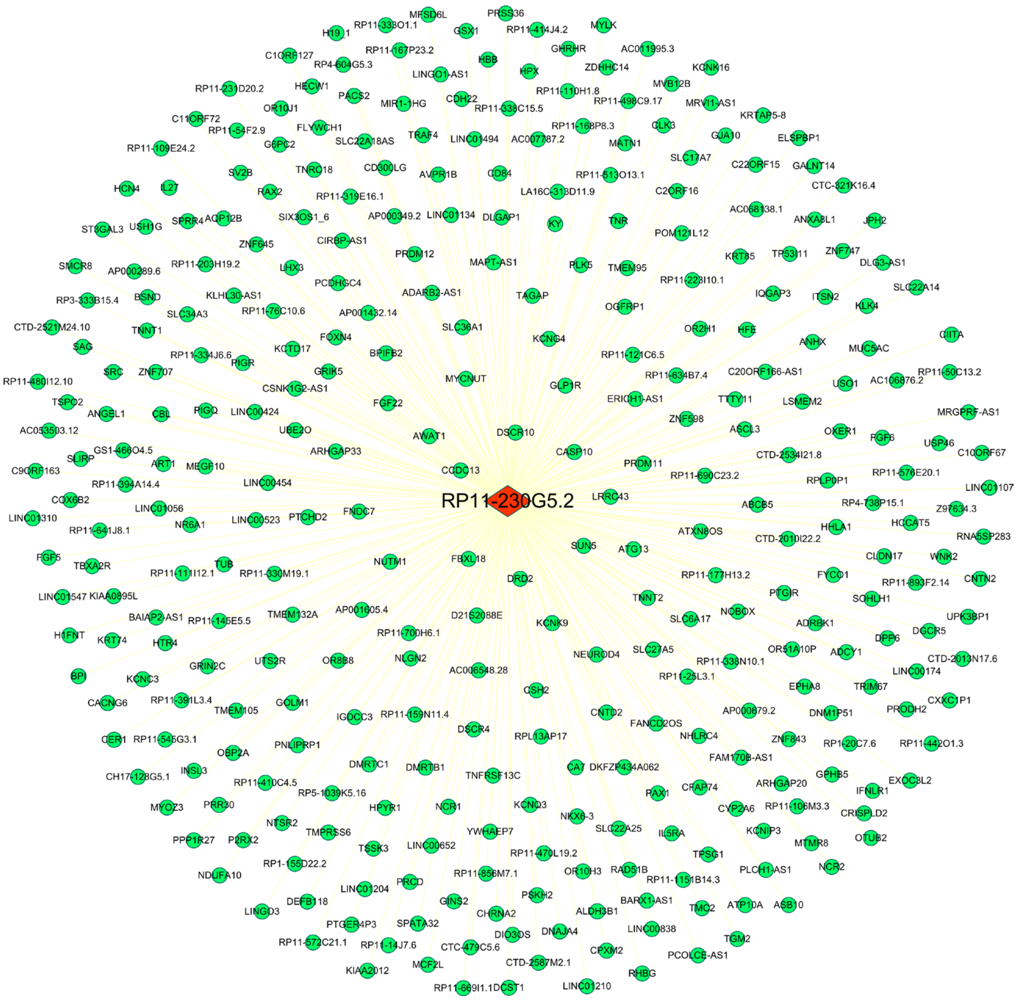

Supplementary Figure 4: Regulation network of lncRNA RP11-230G5.2 by multi experiment matrix.

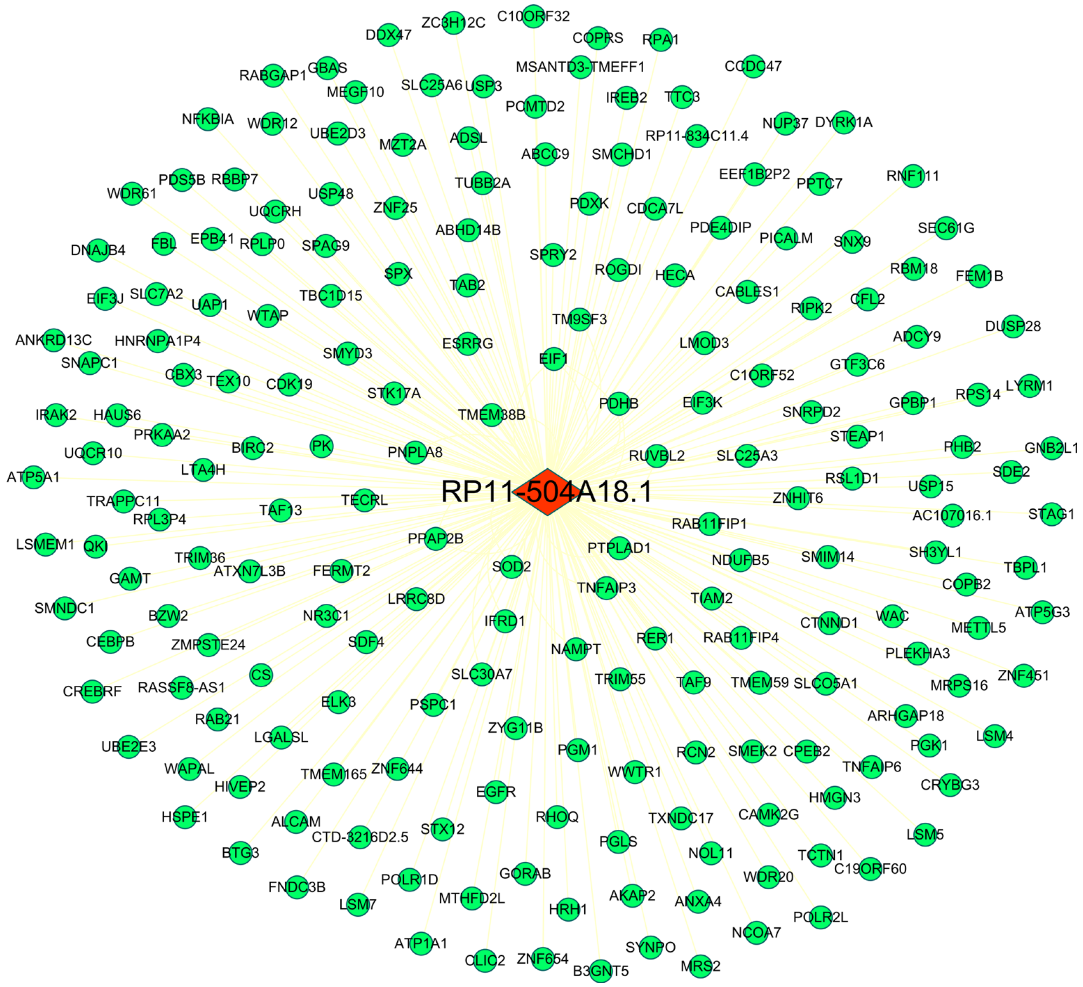

Supplementary Figure 5: Regulation network of lncRNA RP11-504A18.1 by multi experiment matrix.

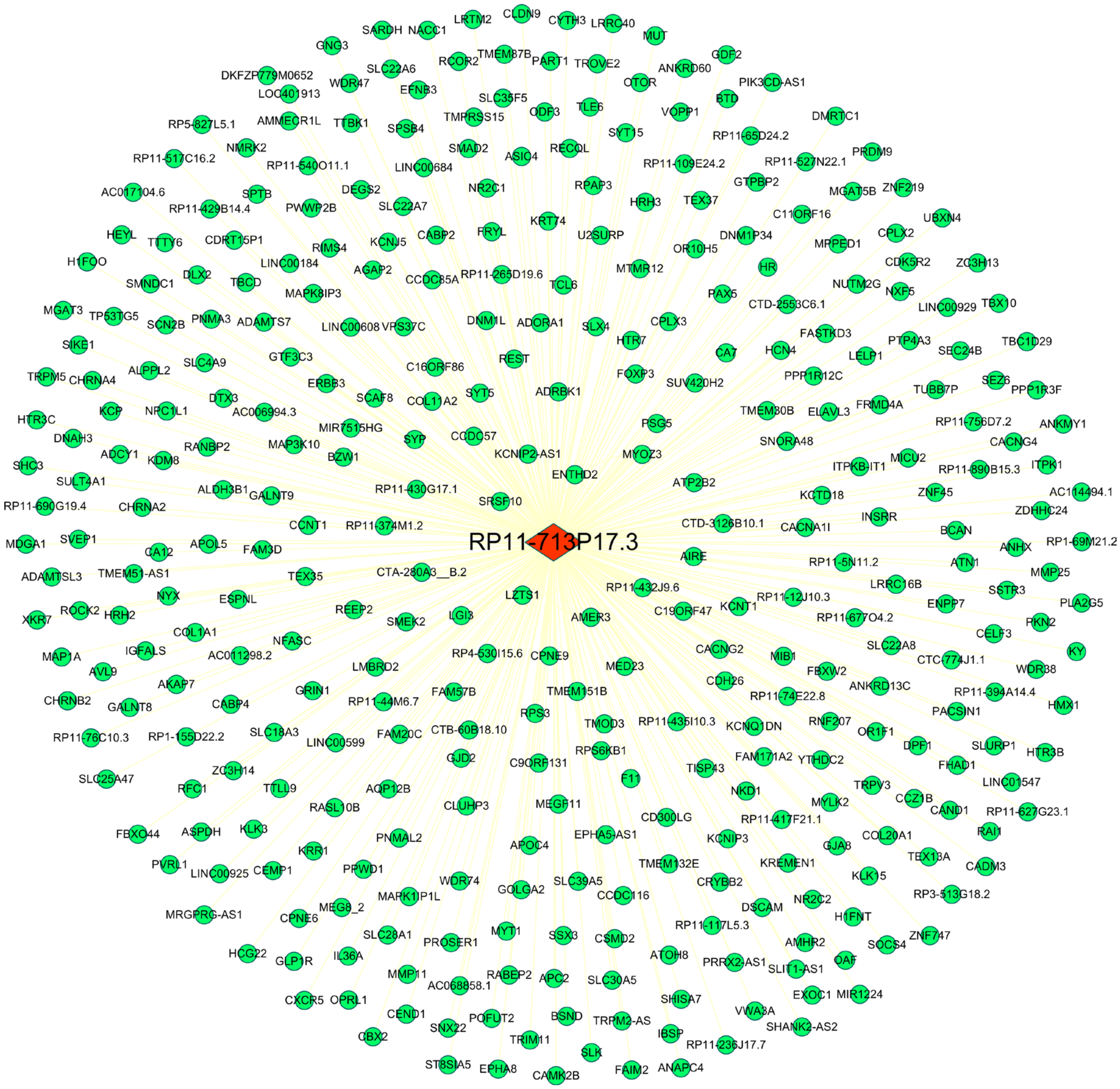

Supplementary Figure 6: Regulation network of lncRNA RP11-713P17.3 by multi experiment matrix.

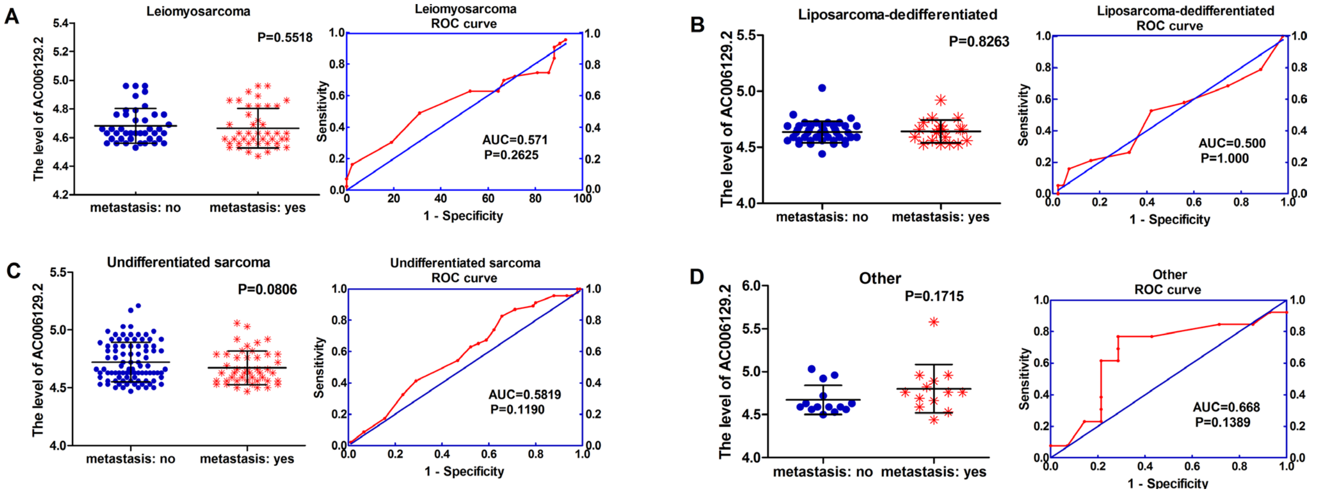

Supplementary Figure 7: Relationship between AC006129.2 level and metastasis in soft-tissue sarcoma based on GEO GSE21050.

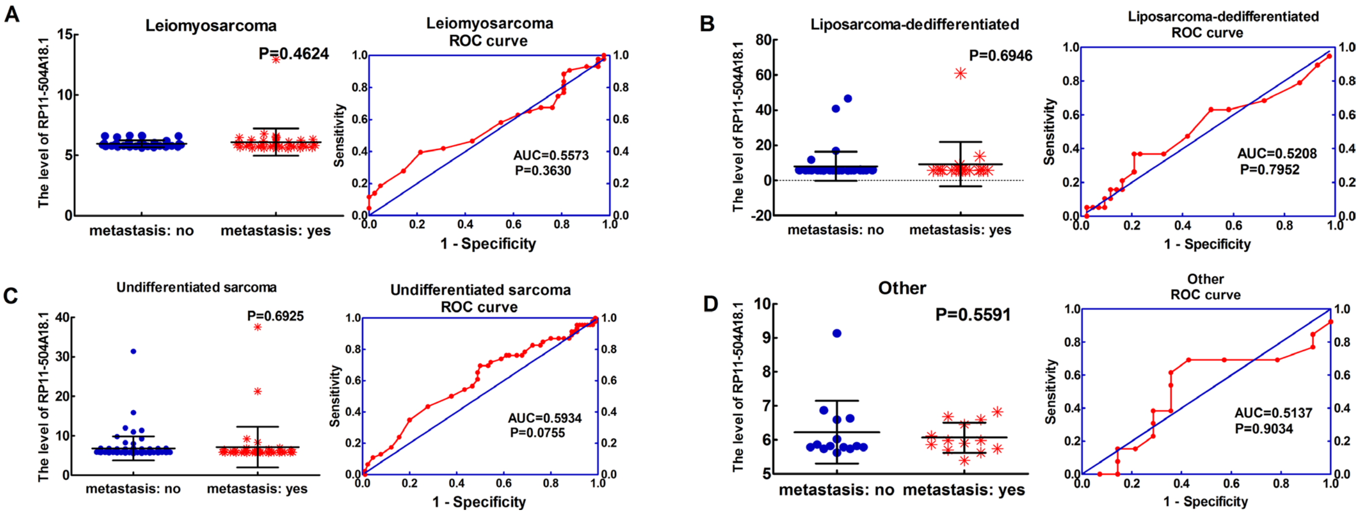

Supplementary Figure 8: Relationship between RP11-504A18.1 level and metastasis in soft-tissue sarcoma based on GEO GSE21050.
